# From Immunodeficiency to Humanization: The Contribution of Mouse Models to Explore HTLV-1 Leukemogenesis

**DOI:** 10.3390/v7122944

**Published:** 2015-12-07

**Authors:** Eléonore Pérès, Eugénie Bagdassarian, Sébastien This, Julien Villaudy, Dominique Rigal, Louis Gazzolo, Madeleine Duc Dodon

**Affiliations:** 1 Laboratoire de Biologie Moléculaire de la Cellule, Unité Mixte de Recherche 5239, Centre National de la Recherche Scientifique, Ecole Normale Supérieure de Lyon, 69364 Lyon Cedex 7, France; eleonore.peres@ens-lyon.fr (E.P.); eugenie.bagdassarian@gmail.com (E.B.); sebastien.this@ens-lyon.fr (S.T.); louis.gazzolo@ens-lyon.fr (L.G.); 2 SFR UMS3444 BioSciences Lyon-Gerland-Lyon Sud (UMS3444), 69366 Lyon Cedex 7, France; 3 Master BioSciences, Département de Biologie, ENS Lyon, 69366 Lyon Cedex 7, France; 4 AIMM Therapeutics, Meibergdreef 59, 1105 BA Amsterdam Zuidoost, The Netherlands; jvillaudy@aimmtherapeutics.com; 5 Department of Medical Microbiology, Academic Medical Center, University of Amsterdam, Meibergdreef 9, 1105 BA Amsterdam Zuidoost, The Netherlands; 6 Etablissement français du sang, 69007 Lyon, France; dominique.rigal@efs-sante.fr

**Keywords:** adult T cell leukemia/lymphoma, HTLV-1, humanized mouse models, oncogenesis

## Abstract

The first discovered human retrovirus, Human T-Lymphotropic Virus type 1 (HTLV-1), is responsible for an aggressive form of T cell leukemia/lymphoma. Mouse models recapitulating the leukemogenesis process have been helpful for understanding the mechanisms underlying the pathogenesis of this retroviral-induced disease. This review will focus on the recent advances in the generation of immunodeficient and human hemato-lymphoid system mice with a particular emphasis on the development of mouse models for HTLV-1-mediated pathogenesis, their present limitations and the challenges yet to be addressed.

## 1. Introduction

Previously known as RNA tumor viruses upon the identification of numerous avian and murine leukemia/sarcoma viruses, retroviruses were thus termed after the discovery of the viral reverse transcriptase in 1970 allowing these viruses to replicate through a DNA intermediate [1,2]. After the description of retroviruses in non-human primates, the long search of human retroviruses ended with the identification of human T-lymphotropic virus type 1 (HTLV-1) and human immunodeficiency virus type 1 (HIV-1) in 1980 and 1983, respectively [3,4].

The description of retroviruses in many species has underlined their broad diversity and revealed their association with numerous diseases encompassing malignant processes, inflammatory disorders and immune dysfunctions. Importantly, retroviruses have participated in the discovery of new cellular and molecular events, opening the field of host-virus interactions in pathological processes. *In vivo* investigations carried out with avian and murine retroviruses inoculated in their natural host (*i.e.*, chickens and mice) have largely contributed to decipher the initiation and development of numerous diseases. Concerning human retroviruses, experimental studies performed *in vitro* with human cells have clarified key events in cell-virus interactions. *In vivo* studies in small (rats, rabbits and mice) and large (monkeys) animals have led to an understanding of transmission, dissemination and persistence of infection.

Since the time of isolation and characterization of human retroviruses, the advent of transgenic and immunocompromised mice has provided investigators with new animal models to apprehend virus-induced diseases. More particularly, immunodeficient mouse strains developing a functional human hemato-lymphoid system (HHLS) after being transplanted with human hematopoietic stem cells (HSC) have been helpful for reaching significant achievements in studying HIV and HTLV-1 related diseases [5,6,7]. Such mouse models fulfill the conditions of reliable animal models ethically acceptable by society, easy to breed at a low cost and convenient to study the pathological processes linked to infection by lymphotropic viruses, such as HTLV-1 [8,9,10,11].

Infection by HTLV-1, a deltaretrovirus, is endemic in Japan, the Caribbean, Western Africa and South and Central America. It is estimated that 10 to 20 million individuals are infected worldwide. Most HTLV-1-infected individuals remain life-long asymptomatic carriers. However, in 3%–5% of cases, HTLV-1 is etiologically linked to a neoplastic syndrome, the adult T cell leukemia/lymphoma (ATLL) and to a spectrum of chronic inflammatory disorders, among which the most frequent is a chronic progressive encephalomyelopathy known as HTLV-1-associated myelopathy/tropical spastic paraparesis (HAM/TSP) [12,13,14].

## 2. The Leukemogenic Activity of HTLV-1

The main clinical feature of ATLL includes leukemic cells with multi-lobulated nuclei called “flower cells” which infiltrate various tissues (skin lesions are very common), abnormal high blood calcium level and opportunistic infections [14]. The CD3+, CD4+, CD8− and CD25+ phenotype of ATLL cells indicates that these cells derive from activated helper T cells. It was reported that in 10 of 17 ATLL cases, leukemic cells express forkhead box P3 (FoxP3), a marker of CD4+ and CD25+ regulatory T (Treg) cells that suppress the proliferation of bystander CD4+ T lymphocytes. Indeed, severe immunodeficiency and complicated opportunistic infections in ATLL patients may arise in part from the immunosuppressive properties of ATLL cells [15,16].

Epidemiological surveys have underlined that ATLL preferentially develops after transmission to neonates through maternal milk. After a prolonged asymptomatic period of 20–40 years, aneuploid leukemic cells emerge. ATLL has been classified into different subtypes: chronic, smoldering, acute and lymphoma. During the long chronic phase of infection, the virus is found integrated in the genome of T lymphocytes (more than 90% are CD4+ T cells). HTLV-1 expression remains undetectable, because of the development of a strong immune response, chiefly mediated by the anti-virus cytotoxic T-lymphocyte response (CTL) [17]. Several HTLV-1-positive CD4+ CD25+ T cell clones that progress from polyclonal to oligoclonal populations are observed. Finally, the outcome of several years of *in vivo* selection results in the dominance of one leukemic clone. At that stage, ATLL patients have a poor prognosis and a median survival time of less than one year. Anti-retroviral treatments, chemotherapies and stem-cell transplantations often fail to cure the disease [18]. Overall, preventing the infection of neonates by HTLV-1 infected mothers remains a crucial issue for the eradication of ATLL [19].

## 3. The Leukemogenic Potential of Tax and HBZ

**Figure 1:**
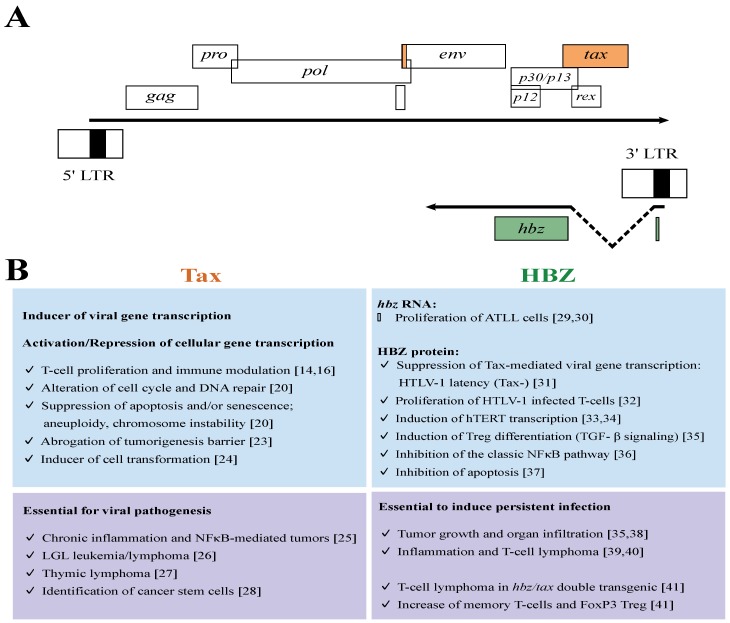
Activities of HTLV-1 Tax and HBZ regulatory proteins *in vitro* and *in vivo*. (**A**) The scheme of HTLV-1 genome showing the sense and antisense genes; these genes are flanked by the long terminal repeats (LTR); (upper part) sense transcripts are initiated in the 5′ LTR containing the promoter region and terminate in the 3′ LTR; (lower part) antisense transcripts are initiated in the 3′ LTR. Coding exons for regulatory proteins are presented as orange box for Tax and green box for HBZ; (**B**) Major roles of Tax and HBZ regulatory proteins reported from experiments using cell culture (upper panel) or transgenic mouse models (lower panel).

The 5′ LTR of the HTLV-1 provirus has been shown to drive sense transcripts that encode structural and regulatory proteins and among the latter the Tax (transactivator of pX) protein [20]. Interestingly, the 3′ LTR of the HTLV-1 provirus drives antisense transcription involved in the translation of another regulatory protein HBZ (HTLV-1 basic leucine zipper factor) [21,22]. Cellular and molecular studies have emphasized that these two HTLV-1 regulatory proteins are exerting a critical role in HTLV-1-induced leukemogenesis (Figure 1).

The Tax protein is known to trans-activate the sense transcription from the 5′ LTR by interacting with members of the ATF/CREB (Activating Transcription Factor/Cyclic AMP Response Element Binding protein) family of transcription factors [23]. Tax is also defined as a modulator of cellular gene expression involved in the proliferation of T lymphocytes mainly via the activation of the NFκB and AP-1 pathways. This protein is able to bypass cell-cycle checkpoints, affects mechanisms involved in the DNA damage response and apoptosis pathways, and is associated with the accumulation of genetic and epigenetic alterations and RNA stability modifications [20].

**Figure 2:**
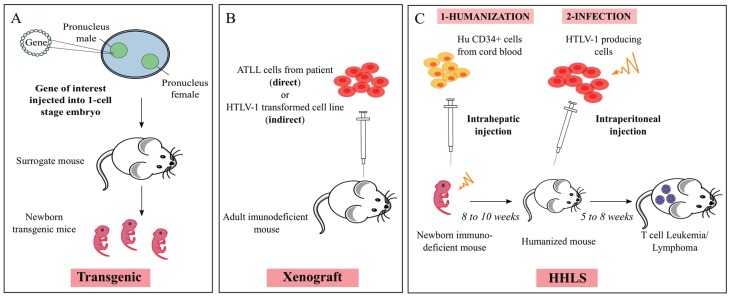
Schematic representation of the protocols used to generate HTLV-1 mouse models. (**A**) Generation of a transgenic mouse model. Briefly, the gene of interest is injected into the male pronucleus of a one-cell embryo. Micro-injected oocytes are introduced into a surrogate female and carried to term. The resulting offspring will then be screened by PCR and sequenced to find the transgenic ones; (**B**) Generation of direct and indirect xenograft mouse models: adult immunodeficient mice are injected either with cells isolated from ATLL patients (direct xenograft) or with HTLV-1 transformed cells (indirect xenograft); (**C**) Generation of Human Hemato-Lymphoid System (HHLS) mouse model: sublethally irradiated newborn immunodeficient mice are engrafted with hematopoietic stem cells. Those humanized mice are then infected with lethally irradiated HTLV-1 producing T cells (see Section 5.2.).

A variety of transgenic mice have been generated to explore the activity of Tax in the initiation and development of HTLV-1-associated diseases [42] (Figure 2A). The first Tax transgenic mice, obtained in 1987 [43], with the *tax* gene being expressed under the control of the HTLV-1 LTR, resulted in the development of multicentric mesenchymal tumors with infiltration of granulocytes. This was the first demonstration that defines Tax as an oncoprotein *in vivo.* These data were later confirmed by the observation of an elevated expression of Tax in bone, associated with aberrant cell functions such as thymic atrophy [44], neurofibromatosis, muscle degeneration, lymphadenopathy, abnormal bone turnover [45] and mesenchymal tumors [25]. Other transgenic mice were generated in which *tax* was placed under the control of different promoters, either viral (Simian virus 40 and Mouse mammary tumor virus) or cellular (CD4, Ig, Granzyme B, Lck, TET, and CD3ε); for a review, see [46]. When *tax* was placed under the control of the Granzyme B (*GzmB*) promoter, which is expressed in mature T cells, transgenic mice exhibit large granular lymphocytic leukemia, associated with splenomegaly and lymphadenopathy, two main clinical features of ATLL [26]. Interestingly, by using a non-invasive imaging of Tax in *GzmB-tax*/LTR-luciferase transgenic mice, inflammation and the subsequent malignancy have been shown to be Tax-dependent through the deregulation of the NF-κB pathway [47]. The constitutive activation of that pathway is essential in the process of Tax-mediated oncogenesis underlining that it constitutes an ideal target for therapeutic treatment and that Tax transgenic mice represent good candidates for preclinical therapeutic *in vivo* trials.

When *tax* was placed under the *Lck* proximal promoter that restricts its expression to developing thymocytes, T cell leukemia with characteristic “flower cells” and lymphoma with infiltrating malignant T lymphocytes highly expressing CD25 are observed [27]. These mice also display a constitutive activation of the NF-κB pathway and a marked hypercalcemia reminiscent to ATLL pathology. Collectively, these observations confirm that Tax expressed in immature thymocytes is sufficient to induce leukemogenesis in transgenic mice.

Yamazaki *et al.* [28] have used a Tax-transgenic mouse model that reproduces ATLL-like diseases. They have observed that the transfer of splenic lymphomatous cells to immunodeficient mice is followed by the regeneration of the original ATLL-like lymphoma. They then detected among lymphomatous cells the presence of a low number of chemotherapy-resistant stem cells. These cells that belong to a minor population of CD38− CD71− CD117+ hematopoietic progenitor cells were shown to be only responsible for the recapitulation of lymphoma in immunodeficient mice. This observation strongly suggests that ATLL leukemic clones exclusively originate in a minor population with stem cell-like properties.

The second regulatory protein HBZ, encoded by the antisense strand of the HTLV-1 provirus, has biologically important activities at both the RNA and protein levels [34]. *hbz* RNA promotes the proliferation of ATLL cells [29], whereas HBZ protein inhibits Tax-mediated viral transcription [21,31]. In addition, HBZ has been shown to modulate the AP-1 [48] and the classical NFκB signaling pathways [36] and to regulate the cell-mediated immune response to virus infection [49]. Nowadays, it is assumed that HBZ is playing an important role in the oncogenic process since it is able to drive infected cell proliferation [30,38], to increase hTERT transcription [33,34] and to inhibit apoptosis [37].

In HBZ transgenic mice, *hbz* RNA promotes CD4+ T cell proliferation [29]. In addition, HBZ protein was found to induce *foxp3* transcription, thus enhancing the number of CD4+FoxP3+ T cells. But a direct interaction between HBZ and FoxP3 proteins leads to an impairment of their regulatory function. Thus, the expression of HBZ in CD4+ T cells appears to be a key mechanism of HTLV-1-induced neoplastic and inflammatory diseases involving interferon-gamma (IFN-γ) [35,39,40]. Moreover, transgenic mice, in which *hbz* is expressed under the control of the CD4 promoter, have been used to test a new vaccine using a recombinant vaccinia virus expressing HBZ. That vaccine was able to induce a cytotoxic memory response against CD4+ T cells expressing HBZ [50]. Finally, double transgenic mice expressing both Tax and HBZ under the control of the CD4 promoter have increased memory T cells and FoxP3+ Treg cells leading to the development of T cell lymphoma and skin lesions [41]. This observation underlines that these two regulatory proteins exert a complementary effect on regulating signaling pathways.

The above observations indicate that the HTLV-1 provirus codes for two main regulatory proteins displaying an oncogenic potential. The question was raised to determine either if they act in a synergistic manner or if they are chronologically involved in the initiation, the maintenance and development of the leukemic process. The latter possibility should be considered since the expression of Tax is frequently disrupted in ATLL cells as indicated by the detection of Tax transcripts in only ~40% of ATLL cases [24]. Analyses of HTLV-1 proviruses and transcripts in ATLL cells revealed three ways in which cells can silence Tax expression: accumulation of nonsense mutations, insertions and deletions in *tax*, DNA methylation of the provirus that silences viral transcription and deletion of the proviral 5′ LTR. The last modification is especially prevalent in acute forms of ATLL. As Tax is the main immunogenic antigen, it is hypothesized that silencing of Tax allows infected cells to escape the CTL response against HTLV-1 [22]. In contrary to Tax, HBZ is expressed all along HTLV-1 infection and HBZ transcription is observed in all ATLL patients [21]. Accordingly, the leukemogenic process may be divided in two phases: the first one under the control of Tax that drives the proliferation of HTLV-1-positive CD4+ CD25+ T cell clones, the second one under the control of HBZ that mediates the proliferation and the maintenance of these clones.

The observations obtained with transgenic mice have provided valuable information about the involvement of these two regulatory proteins in HTLV-1-mediated leukemogenesis. However, these transgenic models do not allow exploring the natural history of HTLV-1 infection, and also the specific intervention of Tax and HBZ during the development of ATLL in humans.

## 4. Mouse Models

### From Immunodeficiency-

**Figure 3:**
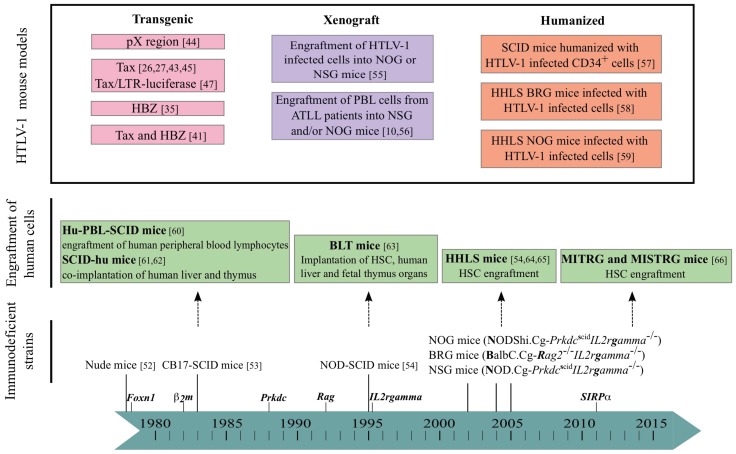
Mouse models in the study of HTLV-1 leukemogenesis. Transgenic immunocompetent mice were mainly used to investigate the role of HTLV-1 Tax and HBZ (see pink boxes). From the beginning of the 1980s, several strains of immunodeficient mice have been isolated and/or developed through the introduction of various gene mutations (in italics over the blue chronological scale). The tumorigenic potential of HTLV-1 infected T-cells or of ATLL cells has been studied by engrafting these cells in immunodeficient mice (xenograft, purple boxes). Likewise, the engraftment of immunodeficient mice with either human lymphocytes or stem cells or lymphoid tissues has led to the generation of humanized mice (green boxes), prone to investigate the role of HTLV-1 infection in leukemogenesis (orange boxes). Hu: humanized; HSC: hematopoietic stem cells; PBL: peripheral blood lymphocytes; HHLS: human hemato-lymphoid system; BLT: bone marrow-liver-thymus; MITRG and MISTRG: M-CSF, IL3, TPO, MG-CSF and/or SIRPα (signal regulatory protein α).

The story of immunodeficient mice strains began fifty years ago with the report of BALB/c *nude* athymic mice that lack a fully developed T cell compartment (Figure 3) [51]. In CB17-SCID (severe combined immunodeficiency) mice, discovered in 1983, mature T and B cells do not develop. Indeed, these mice carry a spontaneous non-sense mutation in the gene coding the protein kinase DNA activated catalytic polypeptide (*Prkdc*), an enzyme necessary for the V(D)J recombination of the B and T cell receptors. However, innate immunity is still functional due to the presence of macrophages, antigen-presenting cells and natural killer (NK) cells [52]. Introducing the SCID mutation onto the non-obese diabetic (NOD) genetic background leads to NOD/SCID mice that display a severe innate immunodeficiency with neither complement system nor functional dendritic cells and macrophages. They provide a good *in vivo* environment for reconstitution with human HSC [53] (see part “to humanization”). In order to avoid thymic education of human HSC on mouse thymus in a MHC(H2)-restricted manner, a targeted mutation into the β*2-microglobulin* (β*2m*) gene was also introduced generating NOD/SCID β*2m^null^* mice lacking the murine immune functions. Later on, a new strain (BALB/c.Cg-*Rag2^null^*) of immunodeficient mice was created by deleting the recombinase-activating gene 2 (*Rag2*) in BALB/c mice [54].

The next generation of immunocompromised mice was obtained by disruption of the IL-2 receptor common gamma chain (γ) gene [67]. These new strains of mice displayed a complete absence of murine T and B cells as well as NK cells. Currently, three major strains of immunodeficient mice are commonly used, NSG (NOD.Cg-*Prkdc^SCID^*-γ*^null^*) [64]; NOG (NODShi.Cg-*Prkdc^SCID^*-γ*^null^*) [65] and BRG (BALB/c.Cg-*Rag2^null^* γ*^null^*) [54]. Their advantages and limitations have been extensively reviewed earlier [5,6,7,68,69].

To improve the human innate immune cell development, MITRG mouse models were developed in which four genes encoding human cytokines (M-CSF, IL3, GM-CSF and TPO) were knocked into their respective mouse loci in *Rag2^null^* γ*^null^* mice. In MISTRG mice, an additional transgene encoding the human signal regulatory protein α (SIRPα) was introduced enabling mouse phagocytes to tolerate and not to phagocyte engrafted xenogeneic cells [66,70].

### -to Humanization

The continuous improvements introduced in creating the immunocompromised mice to favor an efficient engraftment level of human tissue or cells have been exploited to generate humanized mouse models that carry a human functional immune system [10]. In this review, the term “humanized mice” is restricted to severely immunodeficient mice engrafted with human cells and/or tissues and developing a HHLS. These mice have been shown to be valuable to study human immune cell development under normal and pathological conditions.

Two humanized (hu) mouse models hu-PBL-SCID and SCID-hu Thy/Liv were described at the end of the 1980s. The first one is generated through the intraperitoneal inoculation of human peripheral blood lymphocytes (PBL) [60]. The second mouse model is developed after surgical implantation of fetal thymus/liver tissue under the renal capsule of SCID mice to form a conjoint thymus-like organ [61]. Such a model is cumbersome to generate and requires repeated biopsies of the organ. Furthermore, a wasting graft-versus-host disease develops within weeks after implantation of human cells, thus limiting experimentation to a few weeks.

Based on these models, another valuable humanized mouse model called “BLT” (Bone marrow, Liver, Thymus) has been described. NOD/SCID and NSG mice are first implanted with human fetal thymic and liver tissues and then with autologous human HSC. Several weeks later, they show long-term systemic repopulation with human T and B cells, monocytes, macrophages and dendritic cells (DC) [63]. T cells in these mice are educated in the human thymus generating human MHC class I- and II-restricted adaptive immune responses to Epstein-Barr Virus (EBV) infection and are activated by human DCs to mount a potent T cell immune response to superantigens. It represents a convenient model to study many aspects of T cell differentiation and function that could not be studied *in vitro*. But, technical, ethical and logistical reasons render this BLT model complex to carry out limiting its wide usage.

These difficulties sparked interest in the search of new protocols to generate convenient and efficient generations of humanized mice. Materials and methods used to reconstitute the HHLS in mice include many factors, such as choice of human tissue and/or cells, route of inoculation, age and gender of recipient mice and preconditioning regime (irradiation or busulfan) (Figure 2C). Immunodeficient newborn mice such as NSG and BRG mice transplanted with human purified CD34+ cells develop three to four months later a robust HHLS, through T cell thymopoiesis and B cell splenic and bone marrow lymphopoiesis. Interestingly, more T and B cells are found in NSG mice than in BRG ones, showing that NSG mice are more permissive to human cell engraftment than BRG mice. In fact, a reduced phagocytosis of human cells by mouse macrophages was observed in NSG mice. That property may be linked to the mouse SIRPα of the NOD genetic background that better recognizes the “don’t eat me” signal of human CD47 than that of BALB/c background [54,71,72].

The MITRG/MISTRG immunodeficient mice are highly permissive for human cell engraftment and show an efficient development of human innate immune cells such as macrophages and NK cells. However, the increase in human myeloid cells correlated with the presence of human B and T cells at lower frequencies than in NSG mice. As B cells display an immature phenotype, humoral immune responses are low as in other strains of HHLS mice [66]. The development of human red blood cells (RBC) is inefficient, and as macrophages strongly phagocyte mouse RBC, anemia ultimately ensues two to three weeks after engraftment.

## 5. The Mouse Modeling of HTLV-1-Induced Leukemogenesis

The immortalization and transformation of HTLV-1 infected CD4+ T cells have been studied with great limitation in tissue culture and patients. Since ATLL develops through several oncogenic steps in a small percentage of HTLV-1-infected individuals, animal models of ATLL are urgently needed not only to understand the *in vivo* initiation and the progression of the leukemogenic process, but also to perform preclinical studies of potential therapeutic agents. Attempts to reach these objectives have been performed through the use of mouse xenograft models and of HHLS mice. In particular, human T cells in HHLS mice display a phenotype of quiescent/activated and naive/memory cells and appear well suited for exploring HTLV-1 pathogenesis.

### 5.1. Xenogeneic Transplantation Assays

Upon the description of immunocompromised mice, their susceptibility to engraftment with either HTLV-1-infected cell lines or ATLL cells was evaluated (Figure 2B). These experiments confirmed that the engraftment efficacy directly correlated with the abrogation level of the murine immune responses and was dependent on a low NK cell activity, absence of complement activity and impaired macrophage and antigen presenting cell function [53]. Consequently, during the last twenty years, these mice were mainly used as xenogeneic engraftment models to apprehend critical aspects of the multistep development of ATLL [10,73,74]. More particularly, the following observations from three reports underline that the development of xenograft approaches in immunodeficient mice has largely contributed to understand kinetics, metastasis, disease progression as well as the origin of ATLL *in vivo.*

In immunodeficient mice inoculated with HTLV-1 infected MET-1 cells, T cell leukemia with tumors in organs such as liver and kidney and an increase of serum calcium level are observed similar to that in ATLL patients [55]. In these leukemic mice, the increase in serum calcium level correlated with expression of RANK-L (receptor activator of nuclear factor kappa-light-chain-enhancer of activated B cells ligand) and with secretion of parathyroid hormone-related protein and interleukin-6. As MET-1 cells expressed both the adhesion molecules CD11a (LFA-1α) and CD49d (VLA-4α) and produced several matrix metallo-proteinases, these observations underline the importance of these molecules in the spread of ATLL cells.

In the second study, primary ATLL cells from acute or smoldering ATLL patients were intravenously transplanted into neonatal NOD/SCID/β*2m^null^* mice [75]. Acute-type ATLL cells were observed in the peripheral blood and in the lymph nodes of recipients. Engrafted ATLL cells were dually positive for human CD4 and CD25, and displayed patterns of HTLV-1 integration identical to those of donors by Southern blot analysis. These cells infiltrated into recipients’ liver, and formed nodular lesions, recapitulating the clinical feature of each patient. In contrast, in smoldering-type ATLL cases, multiple clones of ATLL cells were efficiently engrafted in NOD/SCID/β*2m^null^* mice. When these clones were retransplanted into secondary NOD/SCID/β*2m^null^* recipients, single HTLV-1-infected clones became predominant, indicating the selection of clones with a dominant proliferative activity.

The third study has addressed the origin of ATLL cells. Nagai *et al.* [56] report that ATLL is sustained by a small population of transformed CD4+ CCR7+ CD45RA+ CD45RO− CD95+ T memory stem (T_SCM_) cells, a unique population with stem cell-like properties, whereas the majority of ATLL cells are CD45RA− CD45RO+ conventional memory T cells. Indeed, in both HTLV-1 carriers and ATLL patients, HTLV-1 provirus was absent in naïve T cells, but was always detected in the three memory (stem, central and effector) subpopulations. *In vitro* culture assays performed with highly purified cells clearly demonstrate that the three memory subpopulations have equal susceptibility to HTLV-1 infection, since they express at least two cell surface receptors for HTLV-1, the heparan sulfate proteoglycans and the VEGF-165 receptor Neuropilin 1 [76]. But among the T memory cells, T_SCM_ cells have a unique potential to self-renew while giving rise to T effector and central memory cells. Such an observation suggests that ATLL is hierarchically organized in the same manner as the normal memory T cell compartment. To further demonstrate the role played by T_SCM_ in the initiation of ATLL, the authors proceeded to xenogeneic transplantation assays and inoculated the three subsets in adult irradiated NOG and NSG mice. They observed that a low number of T_SCM_ cells efficiently repopulated identical ATLL clones and replenish downstream central and effector memory T cells, whereas these two other populations have no such capacities. Taken together, these findings reveal the phenotypic and functional heterogeneity of ATLL cells and identify that the T_SCM_ population is the hierarchical apex of ATLL able to reconstitute identical ATLL clones. This study together with that of Yamazaki *et al.* [28] (see part 3) underline that like other cancers, ATLL may be sustained by a rare population with self-renewal capacity able to support accumulations of genetic abnormalities required for the development of this HTLV-1-induced disease.

Finally, xenogeneic transplantation assays have been performed to define specific therapeutic strategies against dysregulated pathways in HTLV-1-induced pathogenesis. Enhanced survival and reduction of tumor growth can be observed after treatment with inhibitors of NFκB-mediated pathway [77,78], of Bcl-2 family [79] or of histone deacetylase [10]. As HTLV-1 infection leads to genetic alterations, a drug inhibiting double strand break repair has been tested in a xenograft mouse model [80]. Oncolytic therapy using measles virus [81] and antibody therapy blocking CCR4 [82] or CD30 [83] also lead to increased survival in NOD/SCID and NOG mice inoculated with ATLL or HTLV-1 infected cells.

### 5.2. HTLV-1 Infection of Humanized Mice

Faithful recapitulation of ATLL in humanized mice has been challenging but required to further apprehend the natural history of HTLV-1 infection and to approach the importance of the immune response in the development and outcome of ATLL [84]. A first attempt to analyze the molecular and cellular events that control the HTLV-1 induced leukemogenesis was realized by inoculating CD34+ progenitor cells *ex vivo* infected with HTLV-1 in SCID mice engrafted with human fetal thymus and liver tissues [57]. An increased expression of the CD25 marker on thymocytes was observed together with a perturbation of the CD4+ and CD8+ thymocyte subset distribution indicating for the first time that hematopoietic progenitor cells and thymus may be targeted by HTLV-1 in humans. However, HTLV-1 infection of these SCID-hu mice failed to induce oncogenesis. In contrast, as reported by Banerjee *et al.* [85], NOD-SCID mice inoculated with CD34+ cells *ex vivo* infected with HTLV-1 have been shown to develop CD4+ T cell lymphoma. However, inoculation of *ex vivo* infected CD34+ cells might represent a bias since the presence of HTLV-1 infected cells among CD34+ cells in ATLL patients is still a matter of debate [56].

To come closer to the natural infection, we have investigated the *in vivo* effects of HTLV-1 infection in HHLS BRG mice [58]. Newborn mice were engrafted with human CD34+ cells and then infected with lethally irradiated HTLV-1-producing T cells at a time when the three main subpopulations of human thymocytes have been detected, *i.e.*, within a period of one to two months after engraftment (Figure 2C). As soon as three months after infection, significant alterations of human T cell development have been observed, the extent of which correlated with the proviral load. Human T cells from thymus and spleen were activated, as shown by the expression of the CD25 marker, that correlates with the presence of *tax* mRNA and with the increased expression of NFκB dependent genes such as *bfl-1*, an anti-apoptotic gene. Five months after HTLV-1 infection, hepato-splenomegaly, lymphadenopathy and T cell lymphoma/thymoma, in which Tax was detected, were observed in those mice. Thus, *in vivo* HTLV-1 infection of HHLS BRG mice perturbs human thymopoiesis at the level of immature cells, and propels T cell development towards the mature stages [86]. To note that these *in vivo* observations confirm results obtained *in vitro*, showing the ability of Tax to interfere with β-selection, an important checkpoint of early T cell differentiation in the thymus. These data suggest that the infection of immature target T cells in the thymus and the immunodeficient environment of these humanized mice favors the rapid development of a T cell malignancy. Interestingly, these observations suggesting that target cells of the leukemogenic activity of HTLV-1 are recruited among a stem cell population are in line with those showing the role played by T_SCM_ in the initiation of ATLL [56].

Lastly, observations using mice that were generated through a different humanization protocol have been reported [59]. Indeed in that study, sub-lethally irradiated seven-week old NOG mice were submitted to an intra-bone marrow injection (IBMI) of human cord blood CD133^+^ cells. Three to eight months after engraftment, a stable B to T cell ratio was observed in the peripheral blood of these mice indicating the formation of a robust immune system. Four to five months after engraftment, these humanized NOG mice were infected by intra-peritoneal injection of lethally irradiated HTLV-1-producing T cells. Upon infection, the number of human CD4+ T cells in the periphery increased rapidly with the presence of abnormal T cells displaying lobulated nuclei resembling ATLL-specific “flower cells”. Five months later, selective growth of a limited number of human CD25+ infected T cell clones was observed. Interestingly, HTLV-1-specific T cell mediated immune responses were induced in some infected mice, suggesting that an adequate thymic education has occurred in these IBMI-humanized NOG mice. Clearly, it is tempting to speculate that the NOG background may be at the origin of the development of adaptive immune responses. It is interesting to note that both Tax and HBZ are expressed in infected humanized mice. This is reminiscent to what is observed in the early phases of the infection process occurring in patients. However, this dual Tax/HBZ expression persists in humanized mice, probably because of the lack of an efficient immune response. Thus, induction of cellular and humoral immune responses against HTLV-1 in infected IBMI-huNOG mice might represent a valuable approach to investigate the natural history of HTLV-1 infection. It remains to be determined whether this immune response may lead to a down-regulation of Tax expression in these infected mice.

**Figure 4:**
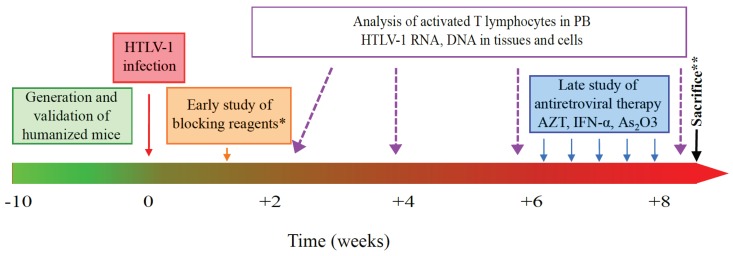
Humanized mice in the development of antiretroviral therapy. A schematic for modeling HTLV-1 infection and therapeutical approaches (orange and blue boxes) in humanized mice. * AZT, NFκB drugs and/or siRNA; ** analysis of activated T lymphocytes, of HTLV-1 DNA and RNA to evaluate the drug efficiency.

Collectively, studies performed to recapitulate HTLV-1 induced leukemogenesis in humanized mice are opening a new chapter in the *in vivo* understanding of pathological mechanisms mediated by the T cell lymphotropic virus. In addition, it is now evident that humanized mice represent a promising preclinical tool to study new therapeutic treatments since the nucleoside analogue reverse transcriptase inhibitor 3′-azido-3′deoxy-thymidine (AZT) was found to be effective at suppressing HIV replication in SCID-hu-mice [62]. Treatments to block the entry or the replication of HTLV-1 could be assessed in order to serve as a post-exposure way to prevent the persistent infection (Figure 4).

Currently, antiretroviral therapies including interferon α (IFN-α), zidovudine (AZT) and As_2_O_3_ have been tested as a first-line therapy for ATLL patients [87]. Furthermore, the anti-CCR4 monoclonal antibody mogamulizumab has been shown to have cytotoxic effects on ATLL cells and is now used in Japan to treat patients [19]. The demonstration of their ability to clear provirus and the understanding of the molecular and cellular mechanisms involved in humanized mice should accelerate clinical approaches for HTLV-1 eradication.

## 6. HHLS Mouse Models and HTLV-1 Pathogenesis: The Future Is Now

The advent of humanized mice to the HTLV-1 research field has offered a challenging opportunity to *in vivo* study ATLL development. Thus far, they have been helpful in elucidating the initial steps of the leukemogenic process induced by this human retrovirus. Concerning HAM/TSP and other immuno-inflammatory disorders associated with HTLV-1 infection, humanized mice have not yet been very useful mainly because of the lack of a strong immune response. One can speculate that enhancement of this immune response through new technologies (see paragraph *iii*) will definitively contribute to a real improvement in the understanding of these pathologies.

Consequently, advances have to be performed to further optimize this mouse model along these three possibilities:

(*i*) To infect humanized mice with molecularly cloned HTLV-1 (unpublished data, Pérès *et al.*) opening a new way not only for understanding in detail the HTLV-1-pathogenesis, but also for delineating the importance of various viral genes on CD4+ T cell transformation and leukemogenesis. Inducible viral gene expression systems could also improve our knowledge [88].
(*ii*) To mimic the way HTLV-1 is delivered (breast-feeding) and disseminated (through dendritic cells) in the body [89]. One can hypothesize that the gastrointestinal tract can serve as a secondary site of infection in which infected T cells present in the milk would be able to infect dendritic cells in the intestine. Clearly, new humanized mouse models engrafted with appropriate target tissues will be suitable for evaluation of HTLV-1 natural infection.
(*iii*) To enhance the specific immune response, by using mouse strains transgenic for human HLAs. For example, in NSG-HLA-A2/HDD mice that possess the human HLA-A2 gene, T cell education is performed in a human HLA context. In these mice, a functional HLA-restricted cytotoxic response has been observed after EBV infection [90]. Likewise, in transgenic NOG/HLA-DR4 mice, T cell homeostasis was differentially regulated in HLA-matched humanized NOG mice compared with HLA-mismatched control mice. Furthermore, antibody class switching was induced after immunization of HLA-DR matched mice with exogenous antigens, underlining that this novel mouse strain will contribute to future studies of human humoral immune responses [91].

Thus, in the near future, it will be possible to infect humanized mice able to develop a fully functional human immune system after transgenic expression of human HLA molecules, cytokines and other species-specific factors and by targeting mouse genes to eliminate host MHC antigens and other genes to further reduce innate immunity [58]. Recently, new technologies for manipulation of the mouse genome have been described (CRISP/Cas9, clustered regularly interspaced short palindromic repeats) and provide exciting opportunities for rapidly generating new genetically modified mice in order to establish a robust small animal model to study the maintenance and development of ATLL [92].

In conclusion, together with observations obtained in immunodeficient mice through transplantation assays, studies performed with HTLV-1-infected mice have documented that the leukemogenic activity of HTLV-1 appears to be dependent on the infection of immature T cells with stem cell-like properties. Even when infected, these rare pre-leukemic cells can generate clonal populations of ATLL cells displaying phenotypic and functional heterogeneity. Therefore, reducing the number of these pre-leukemic cells in HTLV-1 carriers may represent a promising approach to prevent the development of ATLL. In that context, humanized mice would be very useful for testing the chimeric antigen receptor T cell therapy and to eliminate pre-leukemic cells, as recently demonstrated in refractive acute lymphoblastic leukemia [93].
